# The impact of phenotype, ethnicity and genotype on progression of type 2 diabetes mellitus

**DOI:** 10.1002/edm2.108

**Published:** 2020-01-07

**Authors:** Anand Thakarakkattil Narayanan Nair, Louise A. Donnelly, Adem Y. Dawed, Sushrima Gan, Ranjit M. Anjana, Mohan Viswanathan, Colin N. A. Palmer, Ewan R. Pearson

**Affiliations:** ^1^ Population Health & Genomics School of Medicine University of Dundee Dundee UK; ^2^ Madras Diabetes Research Foundation Chennai India

**Keywords:** diabetes progression, glycaemic deterioration, insulin initiation, type 2 diabetes

## Abstract

**Aim:**

To conduct a comprehensive review of studies of glycaemic deterioration in type 2 diabetes and identify the major factors influencing progression.

**Methods:**

We conducted a systematic literature search with terms linked to type 2 diabetes progression. All the included studies were summarized based upon the factors associated with diabetes progression and how the diabetes progression was defined.

**Results:**

Our search yielded 2785 articles; based on title, abstract and full‐text review, we included 61 studies in the review. We identified seven criteria for diabetes progression: ‘Initiation of insulin’, ‘Initiation of oral antidiabetic drug’, ‘treatment intensification’, ‘antidiabetic therapy failure’, ‘glycaemic deterioration’, ‘decline in beta‐cell function’ and ‘change in insulin dose’. The determinants of diabetes progression were grouped into phenotypic, ethnicity and genotypic factors. Younger age, poorer glycaemia and higher body mass index at diabetes diagnosis were the main phenotypic factors associated with rapid progression. The effect of genotypic factors on progression was assessed using polygenic risk scores (PRS); a PRS constructed from the genetic variants linked to insulin resistance was associated with rapid glycaemic deterioration. The evidence of impact of ethnicity on progression was inconclusive due to the small number of multi‐ethnic studies.

**Conclusion:**

We have identified the major determinants of diabetes progression—younger age, higher BMI, higher HbA1c and genetic insulin resistance. The impact of ethnicity is uncertain; there is a clear need for more large‐scale studies of diabetes progression in different ethnic groups.

## Graphical abstract

The aim of this comprehensive review was to identify the determinants of type 2 diabetes progression. Younger age of onset, higher BMI and HbA1c at diabetes diagnosis and genetic insulin resistance were the reported factors associated with diabetes progression.

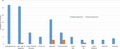

## 1 INTRODUCTION

Type 2 diabetes is a heterogeneous, chronic progressive condition characterized by impaired beta‐cell function and insulin resistance.^1^ The increasing prevalence and rapid progression of diabetes account for the higher cost of the illness and disability‐adjusted life years (DALY).^2^, ^3^, ^4^ In order to prevent or delay the progression of diabetes, or even to target intervention more aggressively to those most likely to rapidly progress, we need to understand the factors associated with the incidence and progression of diabetes.

To date, most research has focused on risk factors for the development of diabetes rather than the progression of diabetes after diagnosis. Lifestyle, obesity and reduced physical activity are important factors associated with diabetes risk.^5^ In addition to these risk factors, there are many biomarkers associated with increased incidence of diabetes. These are classified as glycaemic factors (HbA1c, fructosamine, glycated albumin, 1‐5‐anhydroglucitol), adipose‐derived factors (adiponectin, leptin), hepatic derived factors (alanine aminotransferase, ferritin, insulin‐like growth factor 1), endothelial‐derived factors (cell adhesion molecules, tissue plasminogen activators) and inflammatory factors (c‐reactive protein, interleukin‐6 (IL‐6), interleukin‐13 (IL‐13), interleukin‐17 (IL‐17)).^6^, ^7^, ^8^, ^9^, ^10^ Many studies assessed the role of ethnicity in diabetes incidence, and the Diabetes Study of North California showed higher incidence rates in Asians compared with a White American population.^11^, ^12^, ^13^ Genotypic factors also play an important role in the development of diabetes. There are more than 400 single nucleotide polymorphisms (SNPs) associated with the incidence of diabetes.^14^ The effect of these SNPs is increasingly evaluated by constructing polygenic risk scores (PRS), whereby the individual risk alleles are summed to provide a genetic risk for each individual in a population. Studies of type 2 diabetes PRS showed a significant association with risk of type 2 diabetes after adjustment for other phenotypic factors,^15^, ^16^, ^17^ and a study conducted among Europeans demonstrated a 9.4‐fold risk for individuals in the upper vs lower 2.5% of the PRS.^14^

While it is clearly important to understand diabetes risk factors to prevent the onset of diabetes, it is also important that we identify factors which drive the progression after the onset of diabetes, given the current huge prevalence of diabetes globally. There have been far fewer studies in this critical area. Criteria for diabetes progression after the onset of disease are mainly related to antidiabetic drug requirement (oral hypoglycaemic agents (OHA)s or insulin requirement), drug failure, drug addition, increasing drug dosage, glycaemic level measured as HbA1c, and decline in beta‐cell function. These wide‐ranging definitions reflect a lack of consensus on how diabetes progression is defined and some, but not all of these measures, will be affected by factors related to the patient or prescriber rather than the true underlying progression. That said, if we can recognize the key factors that determine diabetes progression, this will help to identify the fast progressors enabling early intensified treatment and provide biological insights into the process driving progression, which may enable the development of targeted intervention to slow progression rates of diabetes. The objective of this review was to identify and summarize the factors determining type 2 diabetes progression, reflecting glycaemic deterioration, to evaluate the impact of ethnicity on progression and to identify knowledge gaps where further research is required.

## 2 MATERIALS AND METHODS

A comprehensive literature search was conducted in the *MEDLINE (PubMed)* database to identify peer‐reviewed published studies which explored determinants of diabetes progression. In this review, diabetes progression is defined as the progression of diabetes from diagnosis to clinical requirement of insulin or events indicating glycaemic deterioration. The major search terms used were “diabetes mellitus, type 2", “diabetes progression”, “glycaemic deterioration” and “disease progression/epidemiology." These terms were identified from the manuscripts related to type 2 diabetes progression or from the MeSH (Medical Subheading) database of *MEDLINE*. A detailed description of the search terms used is provided in Appendix S1. We included all types of study designs, without any language restriction from the database inception to search date *(01/06/2019)*, in order to capture all relevant studies. Studies across all ethnic groups are included. In addition to searching MEDLINE, we undertook hand searches with cross‐referencing to accommodate all the available studies. We excluded records which were (a) narrative reviews, guidelines or commentaries, (b) studies of type 1 diabetes, (c) studies assessing risk factors for diabetes and progression to diabetes from pre‐diabetes or from a healthy state and (d) studies of progression during or following gestational diabetes mellitus.

### 2.1 Data extraction and analysis

**Figure 1:**
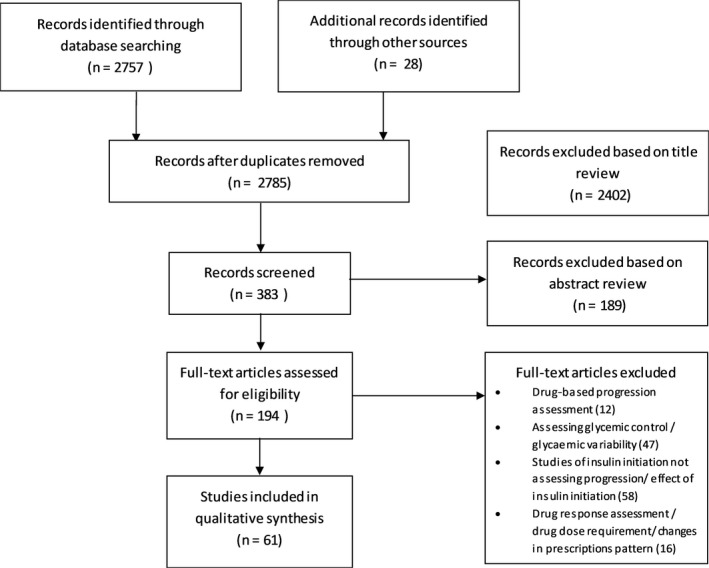
Flow diagram of Literature reduction process

Literature reduction was conducted by reviewing the title, abstract and full paper‐based reviews (Figure 1). From all the included studies, appropriate data was extracted into Excel spreadsheets which included information on author, publication year, study design, sample size, objective of the study, follow‐up period, characteristics of study population, indicator of diabetes progression, country and ethnicity of the study participants, factors affecting diabetes progression and interpretation of study results. The review results have been summarized according to the identified determinants of diabetes progression, design of the study and how the diabetes progression was defined (Table S1).

## 3 RESULTS

The flow diagram of the literature reduction process is provided in Figure 1. Our initial search without any filters provided 2768 records. After removing duplicates, 2757 records remained. The final article pool was created by adding the 28 hand‐searched studies resulting in 2785 records. We reviewed the 2785 titles and selected 383 abstracts based on its relevance to diabetes progression. Based on the abstract review, we retained 194 relevant articles. After the full‐text review, 61 studies were included in the review.

From the 61 included articles, 56 studies assessed the phenotypic factors affecting diabetes progression,^18^, ^19^, ^20^, ^21^, ^22^, ^23^, ^24^, ^25^, ^26^, ^27^, ^28^, ^29^, ^30^, ^31^, ^32^, ^33^, ^34^, ^35^, ^36^, ^37^, ^38^, ^39^, ^40^, ^41^, ^42^, ^43^, ^44^, ^45^, ^46^, ^47^, ^48^, ^49^, ^50^, ^51^, ^52^, ^53^, ^54^, ^55^, ^56^, ^57^, ^58^, ^59^, ^60^, ^61^, ^62^, ^63^, ^64^, ^65^, ^66^, ^67^, ^68^, ^69^, ^70^, ^71^, ^72^, ^73^ while only four studies investigated genotypic determinants of diabetes progression^74^, ^75^, ^76^, ^77^ and one study assessed both phenotypic and genotypic factors.^78^ Before 2000, there were only four studies published and between 2000 and 2010; there were 20 studies published, with the remaining 37 studies published after 2010. The 61 studies were comprised of 35 retrospective cohort studies,^19^, ^20^, ^23^, ^24^, ^26^, ^27^, ^28^, ^29^, ^30^, ^31^, ^34^, ^36^, ^37^, ^38^, ^41^, ^45^, ^49^, ^50^, ^51^, ^53^, ^57^, ^58^, ^60^, ^61^, ^62^, ^63^, ^64^, ^65^, ^67^, ^68^, ^69^, ^70^, ^72^, ^76^ 19 prospective cohort studies,^21^, ^22^, ^32^, ^33^, ^35^, ^39^, ^40^, ^42^, ^43^, ^44^, ^46^, ^48^, ^52^, ^54^, ^56^, ^59^, ^66^, ^71^, ^73^ three cross‐sectional studies,^18^, ^25^, ^47^ three case‐control studies^74^, ^75^, ^77^ studies and one randomized controlled trial (RCT).^79^ The sample size ranged from 50 to 366 955 individuals, and most of the studies were from developed countries with European, American and Australian populations. Further details of the studies are provided in Table S1.

### 3.1 Definitions of “diabetes progression”

There are multiple ways in which investigators have defined diabetes progression. We have identified seven criteria for progression from these studies: (a) “initiation of insulin”: defined as the initiation of first insulin prescription or the start of sustained use of insulin (more than 6 months); (b) “initiation of oral antidiabetic drug”: defined as the initiation of first oral hypoglycaemic agent after the diagnosis of diabetes; (c) “treatment intensification”: defined as the process to attain glycaemic control, characterized by an increase in the dose of OHAs, adding more OHAs or commencing insulin therapy; (d) “antidiabetic therapy failure”: is described as the decline of the effectiveness of OHAs to maintain appropriate glycaemic levels which result in shift to OHA combination therapy or insulin administration; (e) “glycaemic deterioration”: defined as an increase in HbA1c levels after the onset of diabetes (HbA1c > 8% or 1% rise from diagnosis) or rise of coefficient of failure (the coefficient of failure is the slope of the least squares regression line of HbA1c against time)^79^; (f) “decline in beta‐cell function”: pancreatic beta‐cell function is assessed by many methods such as homoeostasis model assessment–beta (HOMA ‐B) or fasting C peptide or proinsulin/insulin ratio; and (g) “change in insulin dose”: an increased requirement of insulin is considered to reflect diabetes progression after the initiation of insulin.

The definition of diabetes progression was broken down in the 61 studies as follows: 34 studies used “initiation of insulin”, ten used “treatment intensification”, seven used “initiation of oral antidiabetic drug”, five used “decline in beta‐cell function”, three used “glycaemic deterioration”, one used “therapy failure” and one used “change in insulin dose.”

### 3.2 Phenotypic determinants of diabetes progression

Among all studies, 57 studies primarily assessed the role of phenotypic factors on the rate of diabetes progression of which 56 studies assessed the role of phenotypic factors only and one study assessed both phenotypic and genotypic factors in regulating the diabetes progression. Despite there being more than 35 biomarkers for the prediction of diabetes incidence, only a few studies have attempted to identify biomarkers for diabetes progression.^10^ The major phenotypic factors associated with diabetes progression across these studies were glycaemia measured as HbA1c or fasting glucose, age of diabetes onset, BMI, gender, diabetes duration, high‐density lipoprotein‐cholesterol (HDL‐C) and triglycerides (TG).

#### 3.2.1 Glycaemia

Twenty‐six studies (20 retrospective cohort studies,^19^, ^24^, ^26^, ^27^, ^29^, ^34^, ^37^, ^38^, ^41^, ^49^, ^51^, ^53^, ^60^, ^61^, ^62^, ^63^, ^64^, ^65^, ^70^, ^78^ five prospective cohort studies^46^, ^48^, ^52^, ^54^, ^71^ and one RCT^55^) reported HbA1c or fasting blood glucose at diagnosis as one of the main determinants of diabetes progression.

The Look AHEAD (Action for Health in Diabetes) trial was conducted to assess the effect of weight loss on the prevention of cardiovascular disease in obese/overweight type 2 diabetes cases. Study participants were randomized into intensive lifestyle intervention or diabetes support education, and the effect on cardiovascular disease was evaluated. A secondary analysis of time to insulin initiation demonstrated an adjusted hazard ratio (aHR) of 1.48 (95% CI 1.36‐1.45) per 1% increase in baseline HbA1c.^55^

From five prospective studies, three used insulin initiation,^46^, ^48^, ^52^ one used beta‐cell dysfunction^54^ and one used secondary diet failure^71^ as the progression marker and all of them showed a significant association between more rapid insulin initiation and higher baseline glycaemia. Three studies with insulin initiation as a marker of diabetes progression reported aHRs ranged from 1.22 to 2.23 per 1% increase in baseline HbA1c.^46^, ^48^, ^52^ The fourth prospective study assessed predictors of beta‐cell stress, using proinsulin/insulin (PI/I) ratio as a surrogate measure. The risk of beta‐cell stress increased by 3.8 times with 1% increase in baseline HbA1c.^54^ The fifth study was the Belfast Diet study which assessed failure of diet therapy. They reported that a lower baseline fasting blood glucose (FBS) was significantly associated with a slower rate of diabetes progression.^71^

Among the 20 retrospective cohort studies, eight assessed insulin initiation, eight reported treatment intensification and four focused on drug initiation. Eight insulin initiation‐based studies estimated the hazard of insulin initiation per 1% increase in baseline HbA1c (%) and aHR varied from 1.09 to 1.33.^19^, ^24^, ^26^, ^41^, ^63^, ^65^, ^70^, ^78^ All eight studies which assessed diabetes progression on the basis of treatment intensification reported baseline HbA1c was significantly associated with rapid treatment intensification.^29^, ^37^, ^38^, ^51^, ^53^, ^60^, ^61^, ^62^ A study conducted in France assessed the hazard of different categories of baseline HbA1c with HbA1c ≤ 7% as a reference in relation to treatment intensification. Hazard ratios for different categories of baseline HbA1c were 7.01%‐8.5% (aHR: 1.51), 8.51%‐9.5% (aHR: 2.16) and ≥9.51% (aHR:1.38). In contrast to the other study findings, there was an unexplained reduction in aHR at the higher HbA1c (%) category compared with other categories.^29^ Of the four studies assessing diabetes progression with antidiabetic drug initiation as a sign of progression, two studies showed higher glycaemia level (fasting blood sugar or HbA1c) was associated with antidiabetic drug prescription.^34^, ^49^ The other two studies reported higher HbA1c levels were associated with shorter time to antidiabetic drug initiation with an aHR of 2.44 (95% CI 1.61‐3.70) for HbA1c greater than 7.5%.^27^, ^64^

**Figure 2:**
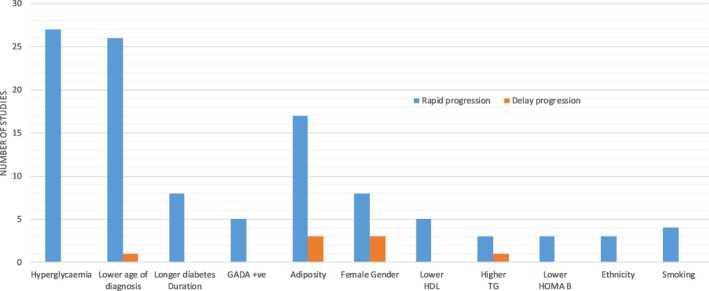
Major factors at diagnosis influencing diabetes progression (factors reported by at least two studies)

Overall, HbA1c at diagnosis is reported as a significant determinant of diabetes progression by 20 retrospective, five prospective and one RCT studies as summarized in Figure 2. HbA1c is a key factor determining diabetes progression: irrespective of the difference in the study population and follow‐up, nearly all studies reported the importance of higher HbA1c with increased risk of diabetes progression.

#### 3.2.2 Age of onset of diabetes and duration of diabetes

A total of 27 studies established the association of younger age of diabetes onset with increased diabetes progression. Of these, 22 were retrospective,^19^, ^23^, ^26^, ^27^, ^29^, ^30^, ^34^, ^37^, ^45^, ^49^, ^50^, ^51^, ^60^, ^61^, ^62^, ^63^, ^64^, ^65^, ^70^, ^72^, ^78^ three were prospective,^43^, ^71^, ^73^ one was a RCT,^55^ and one was a cross‐sectional study.^18^

Analysis of the Look AHEAD trial data showed that younger age of diabetes diagnosis was associated with faster rate of insulin initiation, with an aHR 0.88 (95% CI 0.79‐0.98) per 10 year of age of diagnosis.^55^ In the prospective studies, two studies used time to insulin,^43^, ^73^ and one used diet failure as the progression phenotype,^71^ with all three studies concluding that younger age of diagnosis was associated with faster progression.^43^, ^71^, ^73^

Of the 22 retrospective studies, 10 examined the relationship between time to insulin and age of diabetes^19^, ^23^, ^24^, ^26^, ^30^, ^63^, ^65^, ^70^, ^72^, ^78^ while seven assessed time to treatment intensification,^29^, ^37^, ^45^, ^51^, ^60^, ^61^, ^62^ four assessed the initiation of antidiabetic drug^27^, ^34^, ^49^, ^64^ and one evaluated glycaemic deterioration.^50^ Most of the retrospective studies which considered initiation of insulin as a progression marker reported that a younger age at diagnosis is associated with a higher risk of diabetes progression. While two studies reported age as a risk factor for insulin initiation, of these one study compared the insulin initiation with other antidiabetic injectables and the final study assessed early vs late insulin initiation.^23^, ^26^ A significant association between diabetes progression and age of diabetes was reported by all other longitudinal studies, even though the markers of progression were different. In contrast to the above findings, one cross‐sectional study which used self‐reported information on insulin initiation described that higher age group was associated with early insulin initiation.^18^

Eight studies examined the relationship between time to insulin and diabetes duration, of these six were retrospective^20^, ^23^, ^41^, ^61^, ^65^, ^69^ and two were prospective cohort studies.^46^, ^73^ Diabetes duration is defined as the time between diabetes diagnosis and entry into the study. Most of the studies used insulin initiation as a marker of diabetes progression. Most of these studies reported that participants with longer diabetes duration were more likely to start insulin early compared to those with short duration.

#### 3.2.3 Adiposity‐related factors

One of the main adiposity‐related features associated with diabetes progression was BMI. In some studies, weight or waist circumference was used as an alternative measure of adiposity. There were 20 studies that measured the effect of adiposity on diabetes progression: 13 retrospective,^19^, ^20^, ^34^, ^37^, ^41^, ^49^, ^50^, ^53^, ^60^, ^63^, ^65^, ^67^, ^78^ five prospective,^32^, ^46^, ^48^, ^56^, ^71^ one cross‐sectional^18^ and one RCT.^55^

The Look AHEAD trial reported a 5% increase in hazard of insulin initiation with every 5 kg/m^2^ increase in BMI.^55^ Two of the five prospective studies used weight^71^ and waist circumference^32^ as a measure of adiposity. Two of the prospective cohort studies used insulin initiation as a marker of progression, while secondary diet failure, glycaemic deterioration and beta‐cell dysfunction were used in the other studies. The follow‐up period of these five studies ranged from 2 to 10 years, and most of them were conducted in developed countries. The time to insulin studies showed that with each 1 kg/m^2^ increase in baseline BMI the hazard of insulin initiation increased by between 1% and 8%.^46^, ^48^ Similarly, a cohort study with 131 individuals demonstrated a one inch (2.25 cm) increase in waist circumference was associated with increased risk of progression (aHR 3.13 (95% CI 1.11‐8.91)).^32^ To summarise, four studies showed higher adiposity was associated with rapid progression^32^, ^46^, ^48^, ^71^ and one study reported lower BMI was related to beta‐cell dysfunction.^56^

Among the 13 retrospective studies, seven assessed insulin initiation,^19^, ^20^, ^41^, ^63^, ^65^, ^67^, ^78^ three assessed treatment intensification,^37^, ^53^, ^60^ two assessed initiating antidiabetic drugs^34^, ^49^ and one assessed glycaemic deterioration.^50^ Three time to insulin and treatment intensification‐based studies reported faster progression to insulin in those with higher BMI.^20^, ^37^, ^53^, ^60^, ^63^, ^65^ Contrary to this, a lower BMI was associated with faster progression to insulin in two retrospective studies.^19^, ^41^ These findings are consistent with the IMI‐DIRECT study which demonstrated a “U‐shaped” relationship between diabetes progression (time to insulin) and BMI. This indicates that diabetic individuals with lower and higher levels of BMI progress faster compared with the normal BMI group.^78^ Although the study designs and progression indicators varied across studies, they all suggested baseline obesity as a risk factor for rapid diabetes progression.^18^, ^34^, ^49^, ^50^, ^67^

#### 3.2.4 Gender

In most of the studies, female gender was associated with greater progression to insulin initiation. There were two prospective cohort studies and nine retrospective studies in this group, and most of the studies reported time to insulin initiation. The two prospective studies differed, with one study reporting female gender with increased hazard of insulin initiation compared with men (aHR 2.13, 95% CI 1.08‐4.21),^48^ while the other study reported female gender as protective for insulin initiation (aHR 0.95, 95% CI 0.90‐0.99) with marginal significance.^46^ Two retrospective studies also described female gender as a protective factor for progression,^19^, ^37^ but the seven remaining studies had different findings and reported an aHR of female gender between 1.03 and 1.20 in relation to diabetes progression compared with men.^20^, ^61^, ^63^, ^64^, ^70^, ^72^, ^78^

#### 3.2.5 Lipid‐related factors

HDL‐c, LDL‐c and triglycerides have been reported to be associated with diabetes progression. Five studies assessed the effect of baseline HDL‐c on diabetes progression. Among these five studies, one study was prospective in design, but all studies provided identical results: increased HDL‐c was a protective factor and it delayed the glycaemic deterioration, oral antidiabetic drug initiation or insulin initiation.^40^, ^41^, ^49^, ^50^, ^78^ In addition, the ratio between HDL‐c/apolipoprotein A‐I and M‐HDL‐subclasses also delayed diabetes progression.^40^, ^59^

A significant association was detected between higher LDL‐c and increased diabetes progression in one retrospective study.^41^ The association between baseline triglyceride levels and diabetes progression was investigated by four retrospective studies. Three of them showed an increased risk of progression with increased triglycerides,^34^, ^41^, ^78^ while in one study among a poorly controlled diabetes group, lower triglyceride within a high HbA1c group had rapid insulin requirement relative to the high triglyceride‐lower HbA1c group.^23^ These analyses were adjusted for BMI, but not waist circumference. A 6‐year prospective study conducted among Chinese diabetes cases reported log(TG/HDL‐c) ratio was correlated with beta‐cell dysfunction.^66^

#### 3.2.6 Antibodies and inflammatory markers

Glutamic acid decarboxylase antibodies (GADA) are a marker of autoimmune beta‐cell damage and are one of the major predictors of progression to insulin therapy in type 2 diabetes. Five (one prospective and four retrospective) studies reported that the presence of GADA in type 2 diabetes increased the rate of diabetes progression denoted by insulin requirement^44^, ^57^, ^58^, ^67^ and glycaemic deterioration.^50^ These studies were conducted in European, Japanese and Korean populations. Similarly, the presence of islet‐cell antibodies (ICA) also shortened the time to insulin in type 2 diabetes.^44^ Autoantibodies to protein tyrosine phosphatase isoforms IA‐2 (IA‐2A) were also associated with the time to insulin requirement among type 2 diabetes in the United Kingdom Prospective Diabetes Study (UKPDS)^33^ and a Japanese study^57^; likelihood of insulin treatment almost doubled when GADA and IA‐2 antibodies were present in high titre.^33^ Only one study has looked at inflammatory cytokines. Here, a one‐unit (1 ng/L) increase in interleukin‐6 (IL‐6) levels was associated with a 6% increased risk of insulin therapy over 4 years.^52^

#### 3.2.7 Beta‐cell function

Three prospective studies examined the beta‐cell function by homoeostasis model assessment ‐beta (HOMA‐B). Insulin initiation was the outcome in two studies, and diet therapy failure was the outcome of interest in one study. All three studies concluded that the higher the baseline beta‐cell function, the lower the rate of diabetes progression.^44^, ^52^, ^71^

#### 3.2.8 Other phenotypic factors

A cohort study with 2 years follow‐up showed the association of vaspin with diabetes progression. Vaspin is an adipocytokine which has a potential insulin‐sensitizing effect. Insulin initiation was higher in the lower serum vaspin group indicating the effect of vaspin on progression.^39^ One RCT, two retrospective and one cross‐sectional study evaluated the effect of tobacco smoking on diabetes progression, and all concluded that smoking was significantly associated with the diabetes progression, although it is difficult to separate this effect from other confounding variables such as social class.^18^, ^30^, ^55^, ^78^ Increased fibroblast growth factor 21 (FGF21), a peptide hormone that is potentially associated with weight and glycaemia, was associated with rapid diabetes progression.^21^ Hypertension and family history of diabetes were associated with time to insulin in trial data.^55^ Finally, basal insulin requirement was associated with alanine aminotransferase (ALT) in a cross‐sectional study which assessed insulin requirement.^47^

### 3.3 Ethnicity

Most of the published studies reporting diabetes progression were conducted in White European diabetes populations, with seven studies examining the role of ethnicity on diabetes progression rates. The Look AHEAD trial reported Black (aHR 0.66 95% CI 0.52‐0.83) and Hispanic (aHR 0.77 95% CI 0.63‐0.93) diabetes groups have a slower rate of insulin initiation compared with their white counterparts.^55^ A retrospective study conducted in US veterans assessing the insulin initiation rate in White, Hispanics and Black also showed similar results.^65^ Two studies from Singapore reported Malayas and Indians had higher HbA1c levels relative to Chinese people with diabetes.^48^, ^68^ Another study reported that Asians received antidiabetic prescriptions earlier than Black and Latino counterparts, but this difference was not statistically significant.^49^ Ethnicity was not significantly associated with the initiation of antidiabetic drugs in a retrospective study including Non‐Hispanic white, Asian, African American and Latino ethnicities.^34^ However, in a treatment intensification‐based study, diabetes progression was slower in participants of African American ethnicity compared with the White and Hispanic counterparts.^28^ The limited data available do suggest ethnicity impacts on diabetes progression, but there is a clear research gap in this area.

### 3.4 Genotypic determinants of diabetes progression

Five studies were identified that assessed genotypic factors were associated with diabetes progression.^74^, ^75^, ^76^, ^77^, ^78^ Most of the studies examine the association with progression by developing a polygenic risk score (PRS). A PRS is constructed to aggregate the risk contributed by different genetic variants towards a disease susceptibility. In diabetes progression studies, a PRS derived from diabetes risk variants, a PRS derived from variants related to Beta‐cell function and a PRS derived from variants related to insulin resistance are mostly used (where the Beta‐cell function PRS and insulin resistance PRS include diabetes risk variants that are a subset of the total PRS with evidence of association either with beta‐cell function or with insulin resistance).^80^, ^81^

A case‐control study among those with Japanese ethnicity analysed association between PRS constructed from 11 SNPs associated with reduced beta‐cell function *(rs1111875 in HHEX, rs7756992 in CDKAL1, rs10811661 in CDKN2B, rs13266634 in SLC30A8, rs4402960 in IGF2BP2, rs7903146 in TCF7L2, rs780094 in GCKR, rs7612463 in UBE2E2, rs7172432 in C2CD4A/B, rs2237892 in KCNQ1, and rs5219 in KCNJ11)* and whether they were on insulin therapy. In this crude study of diabetes “progression,” they showed a nominally significant association between this beta‐PRS and insulin treatment (*β* = 0.0131, *SE* = 0.006, *P* = .0431).^74^ Another study among Japanese population explored the association between a single nucleotide polymorphism (SNP) in *Syntaxin IA* and insulin requirement in individuals with type 2 diabetes. The CC genotype had a higher proportion of insulin initiation events compared with the other genotypes, and this study revealed the SNP of S*yntaxin IA* was associated with insulin requirement in a Japanese population. Since the sample size of this study was small (n = 182), the quality of evidence was low.^77^

The IMI‐DIRECT study assessed the association between time to insulin requirement and PRS (derived from 61 type 2 diabetes risk variants). Even though there was no significant association between time to insulin requirement and PRS in the Scottish diabetic population, the PRS was associated with a younger age of diagnosis and a younger age of requirement of insulin.^78^ A study among Caucasians assessed the peroxisome proliferator‐activated receptor (PPAR) alpha gene polymorphism and progression to insulin therapy. The *PPAR‐alpha* variant was significantly associated with time to insulin therapy in the study population, but no replication study has been reported.^75^ In the ADDITION‐Denmark study, the association between a PRS (48 type 2 diabetes genetic risk variants) and time to first prescription (OHA or Insulin) from the diagnosis of type 2 diabetes was evaluated in patients with screen‐detected diabetes. In this study, incident cases were randomized into two intervention groups: a conventional group where the individual received usual care as per national guidelines, and the other which received intensified multifactorial intervention group with lifestyle counselling and additional management for blood pressure and cholesterol. While the overall PRS did not impact on diabetes progression, a sub‐PRS that incorporated only diabetes risk variants linked with insulin resistance showed association with time to insulin prescription (HR 1.39 (95% CI 1.09‐1.77)) in the intensified intervention group.^76^

## 4 DISCUSSION

*Summary of findings*: In this review, we included 61 studies exploring the phenotypic or genotypic determinants of diabetes progression. Major phenotypic factors associated with increased rates of diabetes progression are higher HbA1c at diagnosis, younger age of diabetes onset, higher BMI, lower HDL‐c and higher triglyceride levels at baseline. There were no robust genetic associations, although variants in *PPAR‐alpha*, *Syntaxin IA* and genetic risk scores associated with insulin resistance have been reported to be associated with diabetes progression.

Identification of both phenotypic and genotypic factors associated with diabetes progression will help to recognize those whose glycaemia is likely to progress rapidly and provide intensified treatment, with the aim of reducing the probability of onset of diabetic complications and thereby reducing burden on healthcare systems. Early intervention may also help to tackle the “metabolic memory” where early intensified treatment translates to better long‐term control and lower complication rates.^82^ The majority of the phenotypic studies were conducted in European and American populations with diabetes or other developed countries, but the burden of diabetes is increasing in low‐ and middle‐income countries. There is insufficient evidence to describe the ethnicity‐based differences in diabetes progression; this is most likely to reflect the under‐representation of all diabetes progression studies in non‐white populations.

There was a wide variation in how diabetes progression was defined, with most of the studies defining it as the initiation of insulin^42^, ^43^ with others defining it as the initiation of any OHA or treatment intensification. These prescription‐based analyses can be affected by clinical inertia for prescribing “insulin.” Clinical inertia originates as a result of the complex interaction between patient, provider and health system factors and which delays the appropriate treatment regimes.^83^ The Multinational Diabetes Attitudes Wishes and Needs (DAWN) study representing 13 countries reported the reluctance among healthcare professionals to prescribe insulin.^84^ Similarly from the patient level, cultural and religious beliefs may affect the acceptance of insulin treatment.^85^ Some studies attempted to overcome this limitation by considering both glycaemic levels indicative of insulin requirement and insulin prescriptions but the majority have not.^78^ In addition to this, there is a general prescribing trend of increasing antidiabetic treatment over time, based on the introduction of newer drugs or changes in diabetes management protocol across the countries.^86^ This could have affected the progression assessment based on the initiation of the antidiabetic drug.

Few studies have attempted to analyse the effect of genotypic factors on diabetes progression, and most of the studies were from European and Japanese ethnicities. We need studies with more participants to identify genetic variants or group of genetic variants influencing the diabetes progression.

*Clinical interpretation:* The consensus of the reported studies is that patients were more likely to progress whether they have high HbA1c, are GADA positive and have younger age, high BMI, low HDL‐c and higher triglycerides at baseline. The high HbA1c predicting progression is no surprise and consistent with those having higher HbA1c being most poorly controlled and most likely to fail oral treatment and progress insulin. The presence of GAD antibodies should alert the physician to the increased risk of progression; however, it should be noted that depending on the assay 2.5% to 5% of patients will be GAD positive by chance with this result being a “false positive.” The other parameters reflect a more rapid progression in patients who develop diabetes younger, who are usually more obese and more insulin resistant with adverse lipids. In the extreme, this is described in the TODAY study which reported on type 2 diabetes diagnosed in youth.^87^ In this study, nearly half progressed to the point of treatment failure during the study, and half of these within the first year. How should we treat those at high risk of progression? To date, there are no convincing data that any one drug of the many now available slows progression of diabetes. The ADOPT study seemed to show a slower progression with TZDs compared to sulphonylureas and metformin,^88^ and this is particularly the case with obese women.^89^ Given that, once diabetes has developed, delaying or even stopping glycaemic deterioration in diabetes must be a key goal of diabetes treatment, it is surprising that more studies have not been undertaken to compare progression rates between drugs. It may be that the GRADE study will be of value when reported,^90^ although SGLT2 inhibitors were not included in this study. In the absence of any particular drug to delay progression, clinically we suggest these high‐risk patient subgroups should have regular intensive input from the diabetes multi‐disciplinary team, with a focus on major diet and lifestyle intervention potentially with very low calorie diet^91^ or obesity surgery.^92^

### 4.1 Quality of evidence

Evidence from this review could be affected by the heterogeneity in the study population—a large percentage of studies are conducted among elderly patients with poor glycaemic control and this may introduce bias into the estimations. There were only a few studies which assessed diabetes progression across multi‐ethnic diabetes populations, and this limits our insights into role of ethnicity in diabetes progression. But in all study designs, from prospective to cross‐sectional studies, the factors associated with progression were consistent and large sample size with a longer period of follow‐up in the observational studies provided good quality information. The evidence from this qualitative synthesis could be labelled as “moderate quality” based on the above observations.

In conclusion, the phenotypic and genotypic determinants of diabetes progression identified in this review are glycaemic levels, age of onset of diabetes, BMI and lipid profile; there was no robust genetic association. This review highlights the need for carefully performed multi‐ethnic studies assessing glycaemic deterioration which will help to improve our understanding of diabetes progression. More genetics‐based studies and pooled meta‐analyses are required to validate the current findings on diabetes progression.

## CONFLICT OF INTEREST

None to declare.

## AUTHOR CONTRIBUTIONS

ATN, ERP and LD involved in the design of the review. ATN and ERP determined search strategy and inclusion criteria. ATN drafted the manuscript. ERP, LD, AYD, SG, RMA, VMA and CNP provided review of the manuscript and revised the manuscript.

## ETHICAL APPROVAL

Not required.

## Data Availability

All data related to this analysis are included in the manuscript and supplementary materials.

## References

[1] Fonseca VA . Defining and characterizing the progression of type 2 diabetes. Diabetes Care. 2009;32(suppl_2):S151‐S156 19875543 10.2337/dc09-S301 PMC2811457

[2] Caro JJ , Ward AJ , O'Brien JA . Lifetime costs of complications resulting from type 2 diabetes in the U.S. Diabetes Care. 2002;25(3):476‐481. 11874933 10.2337/diacare.25.3.476

[3] Li R , Bilik D , Brown MB , et al. Medical costs associated with type 2 diabetes complications and comorbidities. Am J Manag Care. 2013;19(5):421‐430. 23781894 PMC4337403

[4] GBD 2015 DALYs and HALE Collaborators NJ , Arora M , Barber RM , et al. Global, regional, and national disability‐adjusted life‐years (DALYs) for 315 diseases and injuries and healthy life expectancy (HALE), 1990–2015: a systematic analysis for the Global Burden of Disease Study 2015. Lancet (London, England). 2016;388(10053):1603‐1658. 10.1016/S0140-6736(16)31460-X PMC5388857 27733283

[5] Sullivan PW , Morrato EH , Ghushchyan V , Wyatt HR , Hill JO . Obesity, inactivity, and the prevalence of diabetes and diabetes‐related cardiovascular comorbidities in the U.S., 2000–2002. Diabetes Care. 2005;28(7):1599‐1603. 15983307 10.2337/diacare.28.7.1599

[6] Dorcely B , Katz K , Jagannathan R , et al. Novel biomarkers for prediabetes, diabetes, and associated complications. Diabetes Metab Syndr Obes. 2017;10:345‐361. 28860833 10.2147/DMSO.S100074 PMC5565252

[7] Sattar N , Wannamethee SG , Forouhi NG . Novel biochemical risk factors for type 2 diabetes: pathogenic insights or prediction possibilities? Diabetologia. 2008;51(6):926‐940. 18392804 10.1007/s00125-008-0954-7

[8] Brahimaj A , Ligthart S , Ghanbari M , et al. Novel inflammatory markers for incident pre‐diabetes and type 2 diabetes: the Rotterdam Study. Eur J Epidemiol. 2017;32(3):217‐226. 28258520 10.1007/s10654-017-0236-0 PMC5380703

[9] Blaha MJ , DeFilippis AP , Rivera JJ , et al. The relationship between insulin resistance and incidence and progression of coronary artery calcification: the Multi‐Ethnic Study of Atherosclerosis (MESA). Diabetes Care. 2011;34(3):749‐751. 21292863 10.2337/dc10-1681 PMC3041221

[10] Abbasi A , Sahlqvist A‐S , Lotta L , et al. A systematic review of biomarkers and risk of incident type 2 diabetes: an overview of epidemiological, prediction and aetiological research literature. PLoS ONE. 2016;11(10):e0163721. 27788146 10.1371/journal.pone.0163721 PMC5082867

[11] Karter AJ , Schillinger D , Adams AS , et al. Elevated rates of diabetes in pacific islanders and Asian subgroups. Diabetes Care. 2013;36(3):574‐579. 23069837 10.2337/dc12-0722 PMC3579366

[12] Anjana RM , Shanthi Rani CS , Deepa M , et al. Incidence of diabetes and prediabetes and predictors of progression among Asian Indians: 10‐year follow‐up of the Chennai Urban Rural Epidemiology Study (CURES). Diabetes Care. 2015;38(8):1441‐1448. 25906786 10.2337/dc14-2814

[13] Sattar N , Gill JMR . Type 2 diabetes in migrant south Asians: mechanisms, mitigation, and management. Lancet Diabetes Endocrinol. 2015;3(12):1004‐1016. 26489808 10.1016/S2213-8587(15)00326-5

[14] Mahajan A , Taliun D , Thurner M , et al. Fine‐mapping type 2 diabetes loci to single‐variant resolution using high‐density imputation and islet‐specific epigenome maps. Nat Genet. 2018;50(11):1505‐1513. 30297969 10.1038/s41588-018-0241-6 PMC6287706

[15] Wu Y , Jing R , Dong Y , et al. Functional annotation of sixty‐five type‐2 diabetes risk SNPs and its application in risk prediction. Sci Rep. 2017;7:43709. 28262806 10.1038/srep43709 PMC5337961

[16] Läll K , Mägi R , Morris A , Metspalu A , Fischer K . Personalized risk prediction for type 2 diabetes: The potential of genetic risk scores. Genet Med. 2017;19:322‐329. 27513194 10.1038/gim.2016.103 PMC5506454

[17] Hara K , Shojima N , Hosoe J , Kadowaki T . Genetic architecture of type 2 diabetes. Biochem Biophys Res Commun. 2014;452(2):213‐220. 25111817 10.1016/j.bbrc.2014.08.012

[18] Abu‐Ashour W , Chibrikova L , Midodzi WK , Twells LK , Gamble J‐MM . Factors associated with early insulin initiation in Type 2 diabetes: a Canadian cross‐sectional study. Diabet Med. 2017;34(2):229‐234. 26802577 10.1111/dme.13082

[19] Donnan PT , Steinke DT , Newton RW , Morris AD . Changes in treatment after the start of oral hypoglycaemic therapy in Type 2 diabetes: a population‐based study. Diabet Med. 2002;19(7):606‐610. 12099966 10.1046/j.1464-5491.2002.00743.x

[20] Nichols GA , Koo YH , Shah SN . Delay of insulin addition to oral combination therapy despite inadequate glycemic control delay of insulin therapy. J Gen Intern Med. 2007;22:453‐458. 17372792 10.1007/s11606-007-0139-y PMC1829438

[21] Ong K‐L , O'Connell R , Januszewski AS , et al. Baseline circulating FGF21 concentrations and increase after fenofibrate treatment predict more rapid glycemic progression in type 2 diabetes: results from the FIELD study. Clin Chem. 2017;63(7):1261‐1270. 28606915 10.1373/clinchem.2016.270876

[22] Perez N , Moisan J , Sirois C , Poirier P , Gregoire J‐P . Initiation of insulin therapy in elderly patients taking oral antidiabetes drugs. CMAJ. 2009;180(13):1310‐1316. 19546456 10.1503/cmaj.080547 PMC2696526

[23] Rigalleau V , Baillet‐Blanco L , Perlemoine C , Salmi L‐R , Gin H . Lower plasma triglycerides are associated with increased need for insulin requirement in poorly controlled Type 2 diabetic patients. Diabet Med. 2005;22(7):877‐881. 15975102 10.1111/j.1464-5491.2005.01548.x

[24] Ringborg A , Lindgren P , Yin DD , Martinell M , Stålhammar J . Time to insulin treatment and factors associated with insulin prescription in Swedish patients with type 2 diabetes. Diabetes Metab. 2010;36(3):198‐203. 20347376 10.1016/j.diabet.2009.11.006

[25] Russo GT , Giorda CB , Cercone S , Nicolucci A , Cucinotta D , BetaDecline Study Group on behalf of BS . Factors associated with beta‐cell dysfunction in type 2 diabetes: the BETADECLINE study. PLoS ONE. 2014;9(10):e109702. 25347846 10.1371/journal.pone.0109702 PMC4210056

[26] Yu M , Mody R , Landó LF , et al. Characteristics associated with the choice of first injectable therapy among US Patients with type 2 diabetes. Clin Ther. 2017;39(12):2399‐2408. 29196084 10.1016/j.clinthera.2017.11.001

[27] Zhang Q , Rajagopalan S , Marrett E , Davies MJ , Radican L , Engel SS . Time to treatment initiation with oral antihyperglycaemic therapy in US patients with newly diagnosed type 2 diabetes. Diabetes, Obes Metab. 2012;14(2):149‐154. 21952003 10.1111/j.1463-1326.2011.01498.x

[28] Ajmera M , Raval A , Zhou S , et al. A real‐world observational study of time to treatment intensification among elderly patients with inadequately controlled type 2 diabetes mellitus. J Manag Care Spec Pharm. 2015;21(12):1184‐1193. 26679967 10.18553/jmcp.2015.21.12.1184 PMC4760631

[29] Balkau B , Bouée S , Avignon A , et al. Type 2 diabetes treatment intensification in general practice in France in 2008–2009: the DIAttitude Study. Diabetes Metab. 2012;38(SUPPL. 3):S29‐S35. 22541600 10.1016/S1262-3636(12)71532-X

[30] Reach G , Le Pautremat V , Gupta S . Determinants and consequences of insulin initiation for type 2 diabetes in France: analysis of the national health and wellness survey. Patient Prefer Adherence. 2013;7:1007‐1023. 24143079 10.2147/PPA.S51299 PMC3797252

[31] Biesenbach G , Raml A , Alsaraji N . Weight gain and insulin requirement in type 2 diabetic patients during the first year after initiating insulin therapy dependent on baseline BMI. Diabetes Obes Metab. 2006;8(6):669‐673. 17026491 10.1111/j.1463-1326.2005.00552.x

[32] Blaha MJ , Gebretsadik T , Shintani A , Elasy TA . Waist circumference, not the metabolic syndrome, predicts glucose deterioration in type 2 diabetes. Obes (Silver Spring). 2008;16(4):869‐874. 10.1038/oby.2008.12 PMC4131716 18277389

[33] Bottazzo GF , Bosi E , Cull CA , et al. IA‐2 antibody prevalence and risk assessment of early insulin requirement in subjects presenting with type 2 diabetes (UKPDS 71). Diabetologia. 2005;48(4):703‐708. 15765222 10.1007/s00125-005-1691-9

[34] Chung S , Zhao B , Lauderdale D , Linde R , Stafford R , Palaniappan L . Initiation of treatment for incident diabetes: evidence from the electronic health records in an ambulatory care setting. Prim Care Diabetes. 2015;9(1):23‐30. 24810147 10.1016/j.pcd.2014.04.005 PMC4221568

[35] Costi M , Dilla T , Reviriego J , Castell C , Goday A . Clinical characteristics of patients with type 2 diabetes mellitus at the time of insulin initiation: INSTIGATE observational study in Spain. Acta Diabetol. 2010;47(Suppl 1):169‐175. 19855919 10.1007/s00592-009-0158-8 PMC3003149

[36] Dale J , Martin S , Gadsby R . Insulin initiation in primary care for patients with type 2 diabetes: 3‐year follow‐up study. Prim Care Diabetes. 2010;4(2):85‐89. 20392683 10.1016/j.pcd.2010.03.001

[37] Fu AZ , Qiu Y , Davies MJ , Radican L , Engel SS . Treatment intensification in patients with type 2 diabetes who failed metformin monotherapy. Diabetes Obes Metab. 2011;13(8):765‐769. 21457427 10.1111/j.1463-1326.2011.01405.x

[38] Fu AZ , Sheehan JJ . Treatment intensification for patients with type 2 diabetes and poor glycaemic control. Diabetes Obes Metab. 2016;18(9):892‐898. 27160505 10.1111/dom.12683

[39] Jian W , Peng W , Xiao S , et al. Role of serum vaspin in progression of type 2 diabetes: a 2‐year cohort study. PLoS ONE. 2014;9(4):e94763. 24732788 10.1371/journal.pone.0094763 PMC3986225

[40] Waldman B , Jenkins AJ , Davis TME , et al. HDL‐C and HDL‐C/ApoA‐I predict long‐term progression of glycemia in established type 2 diabetes. Diabetes Care. 2014;37(8):2351‐2358. 24804699 10.2337/dc13-2738

[41] Gentile S , Strollo F , Viazzi F , et al. Five‐year predictors of insulin initiation in people with type 2 diabetes under real‐life conditions. J Diabetes Res. 2018;2018:1‐10. 10.1155/2018/7153087 PMC6169213 30327785

[42] Nefs G , Pop VJM , Denollet J , Pouwer F . The longitudinal association between depressive symptoms and initiation of insulin therapy in people with type 2 diabetes in primary care. PLoS ONE. 2013;8(11):e78865. 24223860 10.1371/journal.pone.0078865 PMC3815321

[43] Spoelstra JA , Stol RP , de Bruyne MC , et al. Factors associated with switching from oral hypoglycaemic agents to insulin therapy. Neth J Med. 2002;61(9):243‐248. 12365467

[44] Turner R , Stratton I , Horton V , et al. UKPDS 25: autoantibodies to islet‐cell cytoplasm and glutamic acid decarboxylase for prediction of insulin requirement in type 2 diabetes. UK Prospective Diabetes Study Group. Lancet. 1997;350(9087):1288‐1293. 9357409 10.1016/s0140-6736(97)03062-6

[45] Coppell KJ , Lee JE , Williams SM , Mann JI . Progression of glycaemia and cardiovascular risk factors in patients of different age groups with new type 2 diabetes over 5 years of follow‐up in a diabetes quality improvement initiative. Diabetes Res Clin Pr. 2011;93(3):357‐362. 10.1016/j.diabres.2011.04.021 21612837

[46] Danne T , Bluhmki T , Seufert J , et al. Treatment intensification using long‐acting insulin ‐predictors of future basal insulin supported oral therapy in the DIVE registry. BMC Endocr Disord. 2015;15:54. 26446863 10.1186/s12902-015-0051-0 PMC4597397

[47] Kim MK , Jang EH , Son JW , et al. Visceral obesity is a better predictor than generalized obesity for basal insulin requirement at the initiation of insulin therapy in patients with type 2 diabetes. Diabetes Res Clin Pr. 2011;93(2):174‐178. 10.1016/j.diabres.2011.04.009 21565417

[48] Ng T‐P , Goh L‐G , Tan Y , et al. Ethnic differences in glycaemic control in adult Type 2 diabetic patients in primary care: a 3‐year follow‐up study. Diabet Med. 2005;22(11):1598‐1604. 16241927 10.1111/j.1464-5491.2005.01759.x

[49] Pani LN , Nathan DM , Grant RW . Clinical predictors of disease progression and medication initiation in untreated patients with type 2 diabetes and A1C less than 7%. Diabetes Care. 2008;31(3):386‐390. 18083790 10.2337/dc07-1934 PMC3829640

[50] Donnelly LA , Zhou K , Doney ASFF , Jennison C , Franks PW , Pearson ER . Rates of glycaemic deterioration in a real‐world population with type 2 diabetes. Diabetologia. 2018;61(3):607‐615. 29260253 10.1007/s00125-017-4519-5 PMC6448965

[51] Desai U , Kirson NY , Kim J , et al. Time to treatment intensification after monotherapy failure and its association with subsequent glycemic control among 93,515 patients with type 2 diabetes. Diabetes Care. 2018;41(10):2096‐2104. 30131396 10.2337/dc17-0662

[52] Giorda CB , Russo GT , Cercone S , De Cosmo S , Nicolucci A , Cucinotta D . Incidence and correlated factors of beta cell failure in a 4‐year follow‐up of patients with type 2 diabetes: a longitudinal analysis of the BETADECLINE study. Acta Diabetol. 2016;53(5):761‐767. 27193886 10.1007/s00592-016-0868-7

[53] Kallenbach L , Shui AM , Cheng WY , et al. Predictors and clinical outcomes of treatment intensification in patients with type 2 diabetes uncontrolled on basal insulin in a real‐world setting. Endocr Pr. 2018;24(9):805‐814. 10.4158/EP-2017-0261 29975575

[54] Russo GT , Giorda CB , Cercone S , De Cosmo S , Nicolucci A , Cucinotta D . Beta cell stress in a 4‐year follow‐up of patients with type 2 diabetes: A longitudinal analysis of the BetaDecline Study. Diabetes Metab Res Rev. 2018;34(6):1‐9. 10.1002/dmrr.3016 29669179

[55] Pilla SJ , Yeh HC , Juraschek SP , Clark JM , Maruthur NM . Predictors of insulin initiation in patients with type 2 diabetes: an analysis of the look AHEAD randomized trial. J Gen Intern Med. 2018;33(6):839‐846. 29352421 10.1007/s11606-017-4282-9 PMC5975136

[56] Nakayama H , Kato T , Nakayama S , et al. Cross‐sectional and longitudinal analyses of factors contributing to the progressive loss of the beta‐cell function in type 2 diabetes. Intern Med. 2015;54(16):1971‐1976. 26278286 10.2169/internalmedicine.54.4351

[57] Maruyama T , Kasuga A , Ozawa Y , et al. Glutamic acid decarboxylase65 (GAD65) antibodies and insulin auto‐antibodies in Japanese patients with non‐insulin‐dependent diabetes mellitus. Endocr J. 1997;44(1):43‐51. 9152613 10.1507/endocrj.44.43

[58] Hatziagelaki E , Jaeger C , Maeser E , Bretzel RG , Federlin K . GAD 65 antibody but not ICA positivity in adult‐onset diabetic patients is associated with early progression to clinical insulin dependency. Acta Diabetol. 1996;33(4):291‐294. 9033970 10.1007/BF00571567

[59] ‘t Hart LM , Vogelzangs N , Mook‐Kanamori DO , et al. Blood metabolomic measures associate with present and future glycemic control in type 2 diabetes. J Clin Endocrinol Metab. 2018;103(12):4569‐4579. 30113659 10.1210/jc.2018-01165

[60] Yu S , Schwab P , Bian B , Radican L , Tunceli K . Use of add‐on treatment to metformin monotherapy for patients with type 2 diabetes and suboptimal glycemic control: A U.S. Database Study. J Manag Care Spec Pharm. 2016;22(3):272‐280. 27003557 10.18553/jmcp.2016.22.3.272 PMC10397849

[61] Mata‐Cases M , Franch‐Nadal J , Real J , et al. Therapeutic inertia in patients treated with two or more antidiabetics in primary care: Factors predicting intensification of treatment. Diabetes, Obes Metab. 2018;20(1):103‐112. 28656746 10.1111/dom.13045

[62] McEwen LN , Bilik D , Johnson SL , et al. Predictors and impact of intensification of antihyperglycemic therapy in type 2 diabetes (TRIAD). Diabetes Care. 2009;32(6):971‐976. 19228862 10.2337/dc08-1911 PMC2681018

[63] Janghorban M , Amini M . Predictors of switching to insulin from non‐insulin therapy in patients with type 2 diabetes mellitus. Diabetes Res Clin Pract. 2011;92(1):111‐117. 21051096 10.1016/j.diabres.2010.09.033

[64] Sinclair AJ , Alexander CM , Davies MJ , Zhao C , Mavros P . Factors associated with initiation of antihyperglycaemic medication in UK patients with newly diagnosed type 2 diabetes. BMC Endocr Disord. 2012;12(1):1. 22397700 10.1186/1472-6823-12-1 PMC3353844

[65] Parchman ML , Wang CP . Initiation of insulin among veterans with type 2 diabetes and sustained elevation of A1c. Prim Care Diabetes. 2012;6(1):19‐25. 21840276 10.1016/j.pcd.2011.06.006

[66] Zhou M , Li Z , Min R , Dong Y , Sun Q , Li Y . Log (TG)/HDL‐C ratio as a predictor of decreased islet beta cell function in patients with type 2 diabetes: 6‐year cohort study. J Diabetes. 2015;7(5):689‐698. 25327383 10.1111/1753-0407.12229

[67] Lee SA , Lee WJ , Kim EH , et al. Progression to insulin deficiency in Korean patients with Type 2 diabetes mellitus positive for anti‐GAD antibody. Diabet Med. 2011;28(3):319‐324. 21309840 10.1111/j.1464-5491.2010.03186.x

[68] Tan NC , Barbier S , Lim WY , Chia KS . 5‐Year longitudinal study of determinants of glycemic control for multi‐ethnic Asian patients with type 2 diabetes mellitus managed in primary care. Diabetes Res Clin Pract. 2015;110(2):218‐223. 26385596 10.1016/j.diabres.2015.07.010

[69] Irace C , Tripolino C , Carallo C , et al. Clinical predictors of progressive beta‐cell failure in type 2 diabetes. J Investig Med. 2015;63(6):802‐805. 10.1097/JIM.0000000000000210 26057560

[70] Kostev K , Dippel F‐W . Predictors for the initiation of a basal supported oral therapy (BOT) in type 2 diabetic patients under real‐life conditions in Germany. Prim Care Diabetes. 2012;6(4):329‐335. 22749713 10.1016/j.pcd.2012.06.001

[71] Levy J , Atkinson ABB , Bell PMM , McCance DRR , Hadden DRR . Beta‐cell deterioration determines the onset and rate of progression of secondary dietary failure in type 2 diabetes mellitus: the 10‐year follow‐up of the Belfast Diet Study. Diabet Med. 1998;15(4):290‐296. 9585393 10.1002/(SICI)1096-9136(199804)15:4<290::AID-DIA570>3.0.CO;2-M

[72] Machado‐Alba JE , Machado‐Duque ME , Moreno‐Gutierrez PA . Time to and factors associated with insulin initiation in patients with type 2 diabetes mellitus. Diabetes Res Clin Pr. 2015;107(3):332‐337. 10.1016/j.diabres.2015.01.018 25648389

[73] Mast R , Danielle Jansen AP , Walraven I , et al. Time to insulin initiation and long‐term effects of initiating insulin in people with type 2 diabetes mellitus: the Hoorn Diabetes Care System Cohort Study. Eur J Endocrinol. 2016;174(5):563‐571. 26837781 10.1530/EJE-15-1149

[74] Iwata M , Maeda S , Kamura Y , et al. Genetic risk score constructed using 14 susceptibility alleles for type 2 diabetes is associated with the early onset of diabetes and may predict the future requirement of insulin injections among Japanese individuals. Diabetes Care. 2012;35(8):1763‐1770. 22688542 10.2337/dc11-2006 PMC3402252

[75] Flavell DM , Ireland H , Stephens JW , et al. Peroxisome proliferator‐activated receptor alpha gene variation influences age of onset and progression of type 2 diabetes. Diabetes. 2005;54(2):582‐586. 15677519 10.2337/diabetes.54.2.582

[76] Hornbak M , Allin KH , Jensen ML , et al. A combined analysis of 48 type 2 diabetes genetic risk variants shows no discriminative value to predict time to first prescription of a glucose lowering drug in Danish patients with screen detected type 2 diabetes. PLoS ONE. 2014;9(8):e104837. 25157406 10.1371/journal.pone.0104837 PMC4144838

[77] Tsunoda K , Sanke T , Nakagawa T , Furuta H , Nanjo K . Single nucleotide polymorphism (D68D, T to C) in the syntaxin 1A gene correlates to age at onset and insulin requirement in Type II diabetic patients. Diabetologia. 2001;44(11):2092‐2097. 11719842 10.1007/s001250100015

[78] Zhou K , Donnelly LA , Morris AD , et al. Clinical and genetic determinants of progression of type 2 diabetes: a DIRECT study. Diabetes Care. 2014;37(3):718‐724. 24186880 10.2337/dc13-1995 PMC4038744

[79] Wallace TM , Matthews DR . Coefficient of failure: a methodology for examining longitudinal β‐cell function in Type 2 diabetes. Diabet Med. 2002;19(6):465‐469. 12060057 10.1046/j.1464-5491.2002.00718.x

[80] Belsky DW , Moffitt TE , Sugden K , et al. Development and evaluation of a genetic risk score for obesity. Biodemography Soc Biol. 2013;59(1):85‐100. 23701538 10.1080/19485565.2013.774628 PMC3671353

[81] Horne BD , Anderson JL , Carlquist JF , et al. Generating genetic risk scores from intermediate phenotypes for use in association studies of clinically significant endpoints. Ann Hum Genet. 2005;69(Pt 2):176‐186. 15720299 10.1046/j.1529-8817.2005.00155.x PMC4739854

[82] Roberto T , Rita BA , Prattichizzo F , La Sala L , De Nigris V , The CA . “metabolic memory” theory and the early treatment of hyperglycemia in prevention of diabetic complications. Nutrients. 2017;9(5):437. 10.3390/nu9050437 PMC5452167 28452927

[83] Okemah J , Peng J , Quiñones M . Addressing clinical inertia in type 2 diabetes mellitus: a review. Adv Ther. 2018;35(11):1735‐1745. 30374807 10.1007/s12325-018-0819-5 PMC6223992

[84] Peyrot M , Rubin RR , Lauritzen T , et al. Resistance to insulin therapy among patients and providers. Diabetes Care. 2005;28(11):2673‐2679. 16249538 10.2337/diacare.28.11.2673

[85] Lee YK , Lee PY , Ng CJ . A qualitative study on healthcare professionals’ perceived barriers to insulin initiation in a multi‐ethnic population. BMC Fam Pr. 2012;13:28. 10.1186/1471-2296-13-28 PMC3389339 22469132

[86] Wilkinson S , Douglas I , Stirnadel‐Farrant H , et al. Changing use of antidiabetic drugs in the UK: trends in prescribing 2000–2017. BMJ Open. 2018;8(7):e022768. 10.1136/bmjopen-2018-022768 PMC6067400 30056393

[87] Zeitler P , Hirst K , Pyle L , et al. A clinical trial to maintain glycemic control in youth with type 2 diabetes the members of the writing group. N Engl J Med. 2012;24:2247‐2256. 10.1056/NEJMoa1109333 PMC3478667 22540912

[88] Kahn SE , Haffner SM , Heise MA , et al. Glycemic durability of rosiglitazone, metformin, or glyburide monotherapy. N Engl J Med. 2006;355(23):2427‐2443. 17145742 10.1056/NEJMoa066224

[89] Dennis JM , Henley WE , Weedon MN , et al. Sex and BMI alter the benefits and risks of sulfonylureas and thiazolidinediones in type 2 diabetes: a framework for evaluating stratification using routine clinical and individual trial data. Diabetes Care. 2018;41(9):1844‐1853. 30072404 10.2337/dc18-0344 PMC6591127

[90] Nathan DM , Buse JB , Kahn SE , et al. Rationale and design of the glycemia reduction approaches in diabetes: a comparative effectiveness study (GRADE). Diabetes Care. 2013;36(8):2254‐2261. 23690531 10.2337/dc13-0356 PMC3714493

[91] Lean MEJ , Leslie WS , Barnes AC , et al. Primary care‐led weight management for remission of type 2 diabetes (DiRECT): an open‐label, cluster‐randomised trial. Lancet. 2018;391(10120):541‐551. 29221645 10.1016/S0140-6736(17)33102-1

[92] Madsen LR , Baggesen LM , Richelsen B , Thomsen RW . Effect of Roux‐en‐Y gastric bypass surgery on diabetes remission and complications in individuals with type 2 diabetes: a Danish population‐based matched cohort study. Diabetologia. 2019;62(4):611‐620. 30734055 10.1007/s00125-019-4816-2

