# Supplementary material for: The impact of phenotype, ethnicity and genotype on progression of type 2 diabetes mellitus
Source: Endocrinol Diabetes Metab. 2020 Jan 7;3(2):e00108. doi: 10.1002/edm2.108 (PMC7170456; doi:10.1002/edm2.108)
Supplement: Supplementary file 1 [file EDM2-3-e00108-s001.docx]

**Supplementary File 1:**

**Search terms:**

((“Diabetes mellitus, Type 2”[Mesh] OR "Diabetes Mellitus, Type 2/drug therapy"[Mesh] OR "Diabetes Mellitus, Type 2/epidemiology"[Mesh] OR "Diabetes Mellitus, Type 2/genetics"[Mesh] OR "Insulin Resistance"[Mesh] OR NIDDM OR Maturity-Onset Diabetes OR Diabetes Mellitus, Noninsulin-Dependent OR Diabetes Mellitus, Adult-Onset OR Adult-Onset Diabetes Mellitus OR Diabetes Mellitus, Adult Onset OR Diabetes Mellitus, Maturity-Onset OR Diabetes Mellitus, Maturity Onset OR Diabetes Mellitus, Non-Insulin Dependent OR Diabetes Mellitus, Non-Insulin-Dependent OR Non-Insulin-Dependent Diabetes Mellitus OR Diabetes Mellitus, Noninsulin Dependent OR Diabetes Mellitus, Slow-Onset OR Diabetes Mellitus, Slow Onset OR Slow-Onset Diabetes Mellitus OR Diabetes Mellitus, Stable OR Stable Diabetes Mellitus OR Diabetes Mellitus, Type II OR Maturity-Onset Diabetes Mellitus OR Maturity Onset Diabetes Mellitus OR MODY OR Type 2 Diabetes Mellitus OR Noninsulin-Dependent Diabetes Mellitus OR “Type 2 Diabetes”[tiab] OR “Diabetes”[ti])

AND

("Insulin-Secreting Cells"[Mesh]OR "Hypoglycemic Agents"[Mesh] OR "Insulin"[Mesh] OR "Glycated Hemoglobin A"[Mesh] OR "Insulin Resistance"[Mesh] OR HbA1c [All Fields] OR drug dose [All Fields] OR Beta cell [All Fields] OR OHA [All Fields] OR Oral Hypoglycaemic agents [All Fields] )

AND

(“Disease Progression”[Mesh] OR "Disease Progression/drug therapy"[Mesh] OR "Disease Progression/epidemiology"[Mesh] OR "Disease Progression/therapy"[Mesh] OR "Time-to-Treatment"[Mesh] OR “progression” [ti] OR “glycemic deterioration” [ti] OR “glycaemic durability” [ti] OR “glycaemic variability” [ti] OR “HbA1c deterioration” [ti] OR “HbA1c variability” [ti] OR “drug failure” [ti] OR “treatment failure” [ti] OR “drug switch” [ti] OR “drug durability” [ti] OR “insulin initiation” [ti] OR “ insulin requirement” [tiab] OR “insulin delay” [ti] OR “treatment intensification” [ti]))

NOT

("Diabetes Mellitus, Type 1"[MeSH] OR "type 1 diabetes" [tiab] OR "Diabetes Mellitus, Type 2/prevention and control"[Mesh] OR gestational* [All Fields])

***Table S1: Characteristics of the included studies in this review (n=61)***

| **No** | **Author** | **Year** | **Study**  **design** | **Sample size** | **Population** | **Country** | **Major**  **Ethnicity** | **Follow-up**  **period** | **Indicator of progression** | **Relevant Results** |
| --- | --- | --- | --- | --- | --- | --- | --- | --- | --- | --- |
|  | Scott Pilla | 2018 | RCT | 3913 | t2dm | USA | White  American and Black | 10 year | Insulin initiation | Age of diagnosis, ethnicity, HbA1c and BMI was significantly associated with time to insulin |
|  | Guiseppina Russo | 2018 | prospective cohort | 253 | t2dm | Italy | White  European | 4 year | Beta cell dysfunction | High levels of baseline HbA1c was associated with beta cell function deterioration. |
|  | Leen M’t Hart | 2018 | prospective cohort | 2641 | t2dm | Multi country- Europe | White  European | 5 year | Insulin Initiation | ApoA1 and M-HDL-c class was significantly associated with insulin initiation |
|  | Ong, Kwok-Leung | 2017 | prospective cohort | 9697 | t2dm | Australia,  New Zealand | White  Mixed group | 5 years | Initiating Drug | Higher FGF21 concentrations predict more rapid glycemic progression in type 2 diabetes patients |
|  | Mast, R | 2016 | prospective cohort | 2418 | t2dm | Netherlands | White  European | 12 years | Insulin Initiation | Longer diabetes duration (hazard ratio (HR) 1.04 95% CI 1.03–1.05) and lower age (HR 1.00 95% CI 0.99–1.00) at baseline were associated with a shorter time to initiation. |
|  | Danne, T | 2015 | prospective cohort | 31008 | t2dm | Germany | White  European | 3 years | Insulin initiation | Multivariable adjusted hazard ratios demonstrated that longer diabetes duration, higher BMI, poorer glycaemic control, increased the likelihood of BOT initiation |
|  | Hitomi Nakayama | 2015 | prospective cohort | 50 | t2dm | Japan | Japanese | 6.5 year | Beta cell dysfunction | Fasting blood glucose and body mass index found to be associated with beta cell function (6 min post glucagon increment in C-peptide : ΔCPR ) |
|  | Jian, W | 2014 | prospective cohort | 132(t2d)/170 | 132 t2dm | China | Chinese | 2 years | Insulin requirement | The percentage of insulin treatment in T2DM patients was higher in the sub-group with lower serum vaspin level than that in the sub-group with higher vaspin level at follow-up (55.3% vs. 44.7%, P= 0.020) |
|  | Waldman, B | 2014 | prospective cohort | 2608 | t2dm | Australia,  New Zealand | White  mixed | 5 years | Initiating Drug | Prospectively, lower baseline HDL-C and HDL-C/apoA-I levels predicted greater uptake (per 1-SD lower: hazard ratio [HR] 1.13 [CI 1.07– 1.19], P < 0.001; and HR 1.16 [CI 1.10–1.23], P < 0.001, respectively) and earlier uptake of OHAs and insulin. |
|  | Nefs, Giesje | 2013 | prospective cohort | 1389 | t2dm | Netherlands | White  European | 1597 days (mean) | Insulin initiation | The rate of insulin initiation did not differ between depressed and non-depressed participants. |
|  | Costi, M | 2010 | prospective cohort | 224 | t2dm | Spain | White  European | 1 year | Insulin Initiation | Patients with elevated mean HbA1c and body mass index levels had shorter time to insulin initiation |
|  | Giorda,  Carlo B. | 2010 | prospective cohort | 507 | t2dm | Italy | White  European | 4 years | Insulin Initiation | Longer diabetes duration and with higher baseline values for HbA1c, fasting plasma glucose, triglycerides, proinsulin, interleukin-6, HOMA- IR and lower values for HOMA B were associated with insulin administration |
|  | Pérez, Norma | 2009 | prospective cohort | 69674 | t2dm | Canada | White  American | 7 years | Insulin Initiation | Metformine and Thiazide use reduced the progression to insulin therapy |
|  | Blaha, M J | 2008 | prospective cohort | 164 | t2dm | USA | White  American | 2 year (median) | Glycemic deterioration | During follow-up, 39 patients (24%) experienced a hyperglycaemic relapse. The waist circumference was associated with an increased likelihood of hyperglycemic relapse with an aHR of 3.4 (95% confidence interval (CI) 1.2–9.7) |
|  | Bottazzo, G F | 2005 | prospective cohort | 4169 | t2dm | UK | White  European | 6 years | Insulin initiation | IA-2Awere more prevalent in younger The presence of IA-2A together with GADA increased the relative risk of requiring insulin therapy from 5.4 (4.1–7.1) for GADA alone to 8.3 (3.7–18.8) and the corresponding positive predictive value from 33 to 50%. |
|  | Ng, T P | 2005 | prospective cohort | 500 | t2dm | Singapore | Malayas,Indians,Chinese | 3 years | Insulin initiation | A decline in HbA 1c in Malays was significantly less than in the Chinese. Insulin therapy was associated with higher baseline HbA 1c and higher BMI |
|  | Meicen Zhou | 2004 | prospective cohort | 60 | t2dm | China | Chinese | 6 year | Beta cell dysfunction | Higher log (TG)/HDL-C ratio was significantly associated with progressive beta cell dysfunction |
|  | Spoelstra, J.A. | 2002 | prospective cohort | 152 | t2dm | Netherlands | White  European | 4 years | Insulin initiation | A total of 31 (20.4%) patients switched from oral hypoglycaemic agents to insulin therapy; they were significantly younger at the onset and with higher fasting blood glucose levels |
|  | Levy, J | 1998 | prospective cohort | 432 | t2dm | UK | White  European | 10 years | Secondary Diet failure | Continuation on diet alone was associated with a lower ongoing fasting plasma glucose, greater beta-cell function assessed by an oral glucose tolerance test at 6 months, and increasing age |
|  | Turner, R | 1997 | prospective cohort | 1538 | t2dm | UK | White  European | 6 years | Insulin initiation | Presence of GADA and ICA was associated with rapid diabetes progression. |
|  | Donnelly, Louise A | 2018 | retrospective cohort | 5491 | t2dm | UK | White  European | 9.4 years (median follow up) | Glycemic deterioration | The mean glycaemic deterioration for type 2 diabetes and GADA-positive individuals was 1.4 (1.3, 1.4) and 2.8 (2.4, 3.3) mmol/mol HbA1c per year, respectively. Younger age of diagnosis, lower HDL-cholesterol concentration, higher BMI and earlier calendar year of diabetes diagnosis were independently associated with higher rates of glycaemic deterioration. |
|  | Kallenbach Lee | 2018 | retrospective cohort | 14653 | t2dm | USA | White American and Black | 6 months | Treatment intensification | Individuals with hypertension, high HbA1c, and obesity required treatment intensification faster compared to others |
|  | Urvi Desai | 2018 | retrospective cohort | 93515 | t2dm | UK | White  European | 7 year | Treatment Intensification | Higher HbA1c and lower age of diagnosis shortened the time to treatment intensification. |
|  | Manal Mata Cases | 2017 | retrospective cohort | 23678 | t2dm | Spain | White  European | 5 year | Treatment intensification | Higher HbA1c, microvascular complications, and gender were associated with treatment intensification |
|  | Maria Yu, MS | 2017 | retrospective cohort | 11053 | t2dm | USA | White  American | 2 years | Basal Insulin initiation | Patients who were older (adjusted odds ratio [OR], 0.975 [95% CI, 0.971– 0.979]) and had higher HbA1c values (OR, 0.741 [95% CI, 0.721–0.761]) were significantly less likely to be prescribed a GLP-1-RA compared with basal insulin. |
|  | Fu, A Z | 2016 | retrospective cohort | 11525 | t2dm | USA | White  American | 1 year | Treatment intensification | A higher index HbA1c was associated with early intensification. |
|  | Shensheng Yu | 2016 | retrospective cohort | 7109 | t2dm | USA | White  American | 1 year | Treatment intensification | Younger age, Higher HbA1c, Hypertension hyperlipidemia, and obesity was positively associated with treatment intensification |
|  | Chung, S | 2015 | retrospective cohort | 2258 | t2dm | USA | White  American | 1 year | Initiating Drug | Strong positive predictors of drug initiation were younger age, higher fasting glucose at diagnosis, obesity. |
|  | Ngiap Chuan Tan | 2015 | retrospective cohort | 1256 | t2dm | Singapore | Malay, Indian, Chinese | 5 year | Glycaemia deterioration | High HbA1c was associated with insulin. Indians and Malays had glycaemia deterioration compared to Chinese ethnicity. |
|  | Concetta Irace | 2015 | retrospective cohort | 224 | t2dm | Italy | White  European | 6 year | Beta cell dysfunction | Hypertension and longer diabetes duration accelerated the beta cell failure. |
|  | Ajmera, M | 2015 | retrospective cohort | 16653 | t2dm | USA | White  American | 5 years | Treatment intensification | African American ethnicity progressed slower compared to the White and Hispanic counterparts. |
|  | Machado-Alba, Jorge Enrique | 2015 | retrospective cohort | 1042 | t2dm | Colombia | Colombian | 5 years | Insulin Initiation | Being male over 45 years of age (OR: 0.59, 95%CI: 0.37–0.96, p = 0.034) and initiating OAD therapy with metformin (OR: 0.30, 95%CI: 0.20–0.46, p < 0.001) reduced the risk of insulin use |
|  | Hornbak, M | 2014 | retrospective cohort | 1128 | t2dm | Denmark | White  European | 5.7 years (mean follow up) | Initiating Drug | Higher levels of HbA1c, fasting circulating levels of triglyceride, lower HDL, larger BMI and younger age are significant determinants of early pharmacological intervention in type 2 diabetes |
|  | Zhou, K | 2014 | retrospective cohort | 5250 | t2dm | UK | White  European | 8.5 year (mean follow up) | Insulin initiation | Risk of progression was associated with both low and high BMI. Rapid progression was independently associated with younger age at diagnosis, higher log triacylglyceride (TG) and lower HDL concentrations. A high Genetic Risk Score derived from 61 diabetes risk variants was associated with a younger age at diagnosis and a younger age when starting insulin but |
|  | Gérard Reach | 2013 | retrospective cohort | 1933 | t2dm | France | White  European | 3 years | Insulin Initiation | Younger age at diagnosis and current smoking habits were significant predictors of early (versus late) insulin initiation (odds ratio [OR] 1.031, 95% confidence interval [CI] 1.005–1.059, P = 0.0196, and OR 2.537, 95% CI 1.165–5.524, P = 0.0191, respectively). |
|  | Kostev, Karel | 2012 | retrospective cohort | 194967 | t2dm | Germany | White  European | 16 years | Insulin Initiation [Basal] | Predictors of insulin therapy were poor metabolic control, midlife age and number and type of the OAD before insulin use. |
|  | Zhang, Q | 2012 | retrospective cohort | 10743 | t2dm | USA | White  American | 2 years | Initiating Drug | older patients had a greater risk of not receiving treatment with oral antihyperglycaemic therapy than younger patients |
|  | Balkau, B | 2012 | retrospective cohort | 17403 | t2dm | France | White  European | 14 months | Treatment intensification | Treatment intensification was less likely associated with older patients. |
|  | Alan J Sinclair | 2012 | retrospective cohort | 9158 | t2dm | UK | White  European | 2 year | Anti-diabetic drug initiation | Female gender, high HbA1c and younger age of diagnosis are major factors associated with the initiation of anti-diabetic therapy |
|  | Michael L Parchman | 2011 | retrospective cohort | 16480 | t2dm | USA | White  American | 5 year | Insulin Initiation | Higher levels of HbA1c at baseline, younger age non-Hispanic white ethnicity are associated with insulin initiation |
|  | Fu, A Z | 2011 | retrospective cohort | 12566 | t2dm | USA | White  American | 1 year | Treatment intensification | Factors associated with treatment intensification included higher index HbA1c, younger age. |
|  | Coppell K J | 2011 | retrospective cohort | 1108 | t2dm | New Zealand | White  New Zealand | 5 years | Glycemic deterioration | Glycated haemoglobin worsened, especially in the youngest age. |
|  | Gentile, Sandro | 2011 | retrospective cohort | 366955 | t2dm | Italy | White  European | 42 months (median follow up) | Insulin Initiation | Multivariate analysis identified diabetes duration, HbA1c, triglyceride and low HDL-C, LDL-c values were independent predictors of insulin treatment initiation. |
|  | Janghorban, Mohsen | 2010 | retrospective cohort | 6896 | t2dm | Iran | Iranian  (Persian) | 9.3 year (mean follow up) | Insulin Initiation | The switch to insulin from non-insulin therapy was associated with younger age at diagnosis, female gender, higher BMI and HbA1c. |
|  | SA Lee | 2010 | retrospective cohort | 174 | t2dm | South Korea | Korean | 6 year | Insulin Initiation | GADA positive status significantly predicted the progression to insulin deficiency in Korean patients with Type 2 diabetes. In  GADA-positive patients, high-titre GADA and low BMI were associated with this progression. |
|  | Dale, J | 2010 | retrospective cohort | 115 | t2dm | UK | White  European | 3 years | Glycemic deterioration/ insulin initiation | Participants on basal insulin had lesser progression rate and glycemic deterioration. |
|  | A. Ringborg | 2010 | retrospective cohort | 5403 | t2dm | Sweden | White  European | 10 years | Insulin Initiation | The probability of insulin prescription was increased in patients aged less than 65 years (HR= 1.24, 95% CI: 1.03–1.50) HbA1c at the time of starting OAD treatment was also related to the probability of insulin prescription (HR= 1.20, 95% CI: 1.146–1.25) |
|  | Laura n McEwen | 2009 | retrospective cohort | 1093 | t2dm | USA | White  American | 2 year | Treatment intensification | Higher levels of HbA1c and younger age of diabetes diagnosis were associated with treatment intensification |
|  | Pani, L N | 2008 | retrospective cohort | 5804 | t2dm | USA | White  American | 2 years | Initiating Drug | In multivariate analyses, baseline A1C , younger age, and weight gain were independent predictors of progression after adjusting for other covariates. |
|  | Nichols, Gregory A. | 2007 | retrospective cohort | 3891 | t2dm | USA | White  American | 54 months  (mean follow up) | Insulin Initiation | Female sex, Duration of diabetes, Baseline body mass index were independent predictors of insulin use. |
|  | Biesenbach, G | 2005 | retrospective cohort | 192 | t2dm | Austria | White  European | 1 year | Insulin dose | The insulin requirement increased by 22% in the normal- weighted group and by 23% in both groups with overweight. |
|  | Rigalleau, V | 2004 | retrospective cohort | 103 | T2dm | France | White  European | 5 years | Insulin Initiation | The long duration of diabetes, a high HbA1c, and a normal triglyceride level were associated with the need for insulin |
|  | Donnan, P. T | 2002 | retrospective cohort | 1305 | t2dm | Scotland | White  European | 588 days (median follow up) | Insulin Initiation | Independent predictors of insulin initiation were associated with being younger, male, having low BMI and higher HbA1c |
|  | Taro Maruyama | 1997 | retrospective cohort | 229 | t2dm | Japan | Japanese | 5 year | Insulin Initiation | Among GAD positive group 17.6% insulin initiation reported while GAD negative group it was 2.4 % only and the difference was statistically significant. |
|  | E Hatziagelaki | 1996 | retrospective cohort | 150 | t2dm | Germany | White  European | 5 year | Insulin Initiation | GAD positive diabetic cases had insulin requirement more compared to GAD negative diabetes cases. |
|  | Minoru Iwata | 2012 | case control | 734 | t2dm | Japanese | Japanese | - | Insulin Initiation | Among the subjects with type 2 diabetes, the b-GRS was associated with individuals receiving insulin therapy (b = 0.0131, SE = 0.006, P = 0.0431) |
|  | Flavell, D M | 2005 | case control | 912 | t2dm | UK | White  European | - | Insulin | Intron 1 C-allele carriers also progressed more rapidly to insulin monotherapy |
|  | Tsunoda, K | 2001 | case control | 182 | t2dm | Japanese | Japanese | - | Insulin initiation | Individuals with the CC genotype had a higher frequency of insulin treatment (78.30% vs 46.80%, p = 0.006) with a duration equal to, or longer than, 10 years |
|  | Russo, Giuseppina T | 2014 | cross sectional | 507 | t2dm | Italy | White  European | - | Beta cell dysfunction | In multivariate analysis, the risk of having a P/I ratio in the upper quartile was higher males (OR 1.8; 95% CI, 1.1–2.9) |
|  | Abu-Ashour, W. | 2012 | cross sectional | 302700 | t2dm | Canada | White  European | - | Insulin initiation | Age, smoking status, BMI, education, and oral antidiabetic medication use associated with of insulin initiation. |
|  | Kim, M K | 2011 | cross sectional | 50 | t2dm | Korea | Korean | - | Insulin initiation | Multiple linear regression analysis showed that visceral fat area, HbA1C, and ALT are independent predictors of basal insulin requirement |
